# Supplementary material for: Neurocognitive analyses reveal that video game players exhibit enhanced implicit temporal processing
Source: Commun Biol. 2022 Oct 11;5:1082. doi: 10.1038/s42003-022-04033-0 (PMC9553938; doi:10.1038/s42003-022-04033-0)
Supplement: Supplementary file 2 — Supplementary Information [file 42003_2022_4033_MOESM2_ESM.pdf]

Neurocognitive analyses reveal that video game players exhibit enhanced implicit temporal processing

Francois R. Foerster [1]
Matthieu Chidharom [1,2]
Anne Bonnefond [1]
Anne Giersch[1]

[1] Université de Strasbourg, INSERM U1114, Pôle de Psychiatrie, Centre Hospitalier Régional Universitaire de Strasbourg, , France

[2] Department of Psychology, Lehigh University, Bethlehem, PA, USA

Supplementary Information

Supplementary Note 1: Anticipated errors in the variable foreperiod task.
VGPs are believed to be impulsive (but see [1]), and we first verified a possible difference between VGPs and NVGPs on anticipation errors. We compared the number of premature responses (anticipation errors – responding before the target appearance) across groups and cueing conditions. A three-way repeated-measures ANOVA (rANOVA) with the factors Group, Cue and Foreperiod revealed main effects of the Foreperiod $F(1,44 = 23.57$, $p < .0001$; $\eta^2_p = .359$) and the Cue ($F(1,44 = 9.11$, $p = .003$; $\eta^2_p = .171$). In all participants there was less anticipations in trials with short rather than long FP ($Mean_{ShortFP} = 2.48$, $CI_{ShortFP} = 0.81$; $Mean_{LongFP} = 6.43$, $CI_{LongFP} = 1.69$) and less in the neutral rather than the temporal cue condition ($Mean_{Neutral} = 3.48$, $CI_{Neutral} = 0.91$; $Mean_{Temporal} = 5.39$, $CI_{Temporal} = 1.46$). Also, the analysis revealed a main effect of the Group ($F(1,44 = 4.17$, $p = .047$; $\eta^2_p = .087$), indicating less anticipation errors in VGPs than in NVGPs ($Mean_{VGPs} = 6.83$, $CI_{VGPs} = 2.32$; $Mean_{NVGPs} = 10.91$, $CI_{NVGPs} = 3.34$). No interaction effects were revealed (all $p > .303$). As previously reported [2], our analysis does not evidence more motor impulsivity in VGPs than in NVGPs, but rather suggest a reduced motor impulsivity in VGPs. Anticipation errors (1.85 % of the data) and response times that were more than two standard deviations from the mean of each participant (Mean per participant = 3.5 % of the data, SD = 1.85 %) were then discarded from further analyses.

Supplementary Note 2: additional analyses of behavioral data.
A control analysis was conducted to evaluate the possibility that reaction times performance was optimized faster over the course of the experiment in VGPs compared with NVGPs. To do so, a two-way repeated-measures ANOVA with the factors Group and Block was performed on the reaction times data. The analysis revealed a main effect of the Block ($F(3, 132) = 10.718$, $p < .0001$, $\eta^2_p = .196$). However, post-hoc analyses did not reveal significant differences between trial blocks (all $p > .095$). Crucially, no main effect of the Group (p = .16) nor interaction effect (p = .065) was reported (see Supplementary Fig. 1B). Hence, the improvement of the performance over the course of the experiment appears non-specific to the group of participants.

An additional behavioral analysis evaluated specifically the learning of the foreperiods. The total of 480 trials was divided in 10 arbitrary sub-blocks of 48 trials, independently of the type of cue. The implicit benefit from the passage of time was estimated for each of these sub-blocks. According to the learning to learn account [3–5], VGPs might have improved their benefit from the passage of time over the course of the experiment at a faster pace than NVGPs. A two-way repeated-measures ANOVA with the factors Group and Sub-block was performed on the benefit from the passage of time values. The analysis revealed a main effect of the Sub-block ($F(3, 132) = 132.718$, $p < .0001$, $\eta^2_p = .196$). Tukey post-hoc tests (45 comparisons) revealed only two significant comparisons, with participants benefiting more from the passage of time during sub-block 1 ($p = .0421$) and 2 ($p = .0191$) compared to sub-block 7. No other main effect of the Group ($p = .161$) or interaction effect ($p = .065$) was reported. Thus, the analysis suggests a decrease (rather than an increase) of the benefit from the passage of time over the course of the experiment independently of the groups (Supplementary Fig. 1C). This suggests an effective learning of the foreperiod during the training (pre-test) phase. Given the between-block design of the factor Cue (i.e. the order of the blocks with the neutral and temporal cues conditions varied across participants), such an analysis could not be performed to evaluate the learning of the cues.

A recent framework proposed that variability in timed behaviour reflects the efficiency of timing mechanisms [6]. Based on raw reaction time data (Supplementary Fig. 1A), an exploratory analysis investigated their variability across groups, foreperiods and cueing conditions (Supplementary Fig. 1D), most likely reflecting the learning of the foreperiod and the usage of the temporal cue. A three-way repeated-measures ANOVA with the factors Group, Foreperiod and Cue performed on the standard deviation (SD) revealed a main effect of the Group ($F(1, 44) = 6.78$, $p = .012$, $\eta^2_p = .134$), the Cue ($F(1, 44) = 13.08$, $p = .0007$, $\eta^2_p = .229$) and an interaction effect between the Group and the Cue ($F(1, 44) = 5.081$, $p = .029$, $\eta^2_p = .104$). The reaction times appear more stable in VGPs (Mean = 43, CI = 6) than NVGPs (Mean = 54, CI = 7). Also, participants' reaction times were more stable in trials with the neutral cue (Mean = 47, CI = 5) rather than the temporal cue (Mean = 50, CI = 6), possibly reflecting some fluctuations in the usage of the temporal cue. Post-hoc analysis of the interaction effect did not reveal significant differences (all $p > .09$).

To test the reliability of the non-significant findings in behavioral data analysis reported in the manuscript, we performed an additional Bayesian repeated-measure ANOVA [9] using Jamovi (v.1.6). Here, we used a model comparison approach. Within the Bayesian framework, the strength of the evidence supporting H0 or H1 is reflected in the value of the Bayes Factor (BF_{10}).

First, we evaluated the evidence for or against the absence of effect of the Trial Block variable on the benefit from the passage of time. The analysis reports weak to moderate evidence for the absence of a main effect of the Trial Block (BF_{10}= 0.223), for the absence of interaction effects between the Trial Block and the Group (BF_{10}= 0.369), between Trial Block and the Cue (BF_{10}= 0.210), and for the absence of triple interaction effect between the Trial Block, the Group and the Cue (BF_{10}= 0.349).

Second, we evaluated the evidence for or against the absence of effect of the Trial Block variable on the benefit from the temporal cue. The analysis reports weak evidence for the absence of a main effect of the Trial Block (BF_{10}= 0.557), for the absence of interaction effects between the Trial Block and the Group (BF_{10}= 0.283), between Trial Block and the Foreperiod (BF_{10}= 0.243), and for the absence of triple interaction effect between the Trial Block, the Group and the Foreperiod (BF_{10}= 0.294). Overall, all analyses suggest an absence of the effect of the trial block on behavioral data.

Supplementary Fig. 1: Behavioural data.

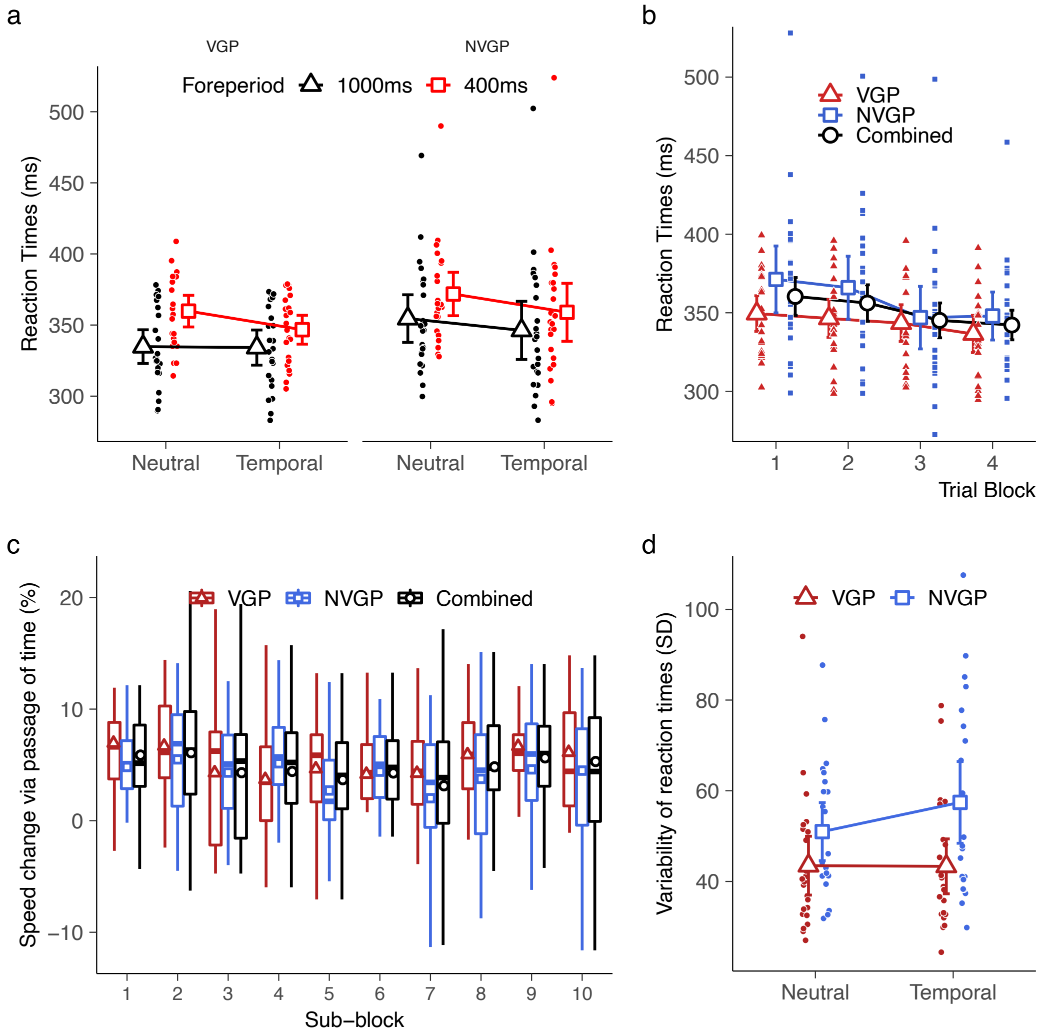

a Reaction times across Group, Cue and Foreperiod. There was an outlier in the NVGP group, who was slower than all other participants (but the difference in responses between conditions was similar to the one found in other participants). Removing the NVGP outlier did not affect the statistical analysis. **c** Participants increased their reaction time over the course of the experiment (B). Benefit from the passage of time over the course of the experiment. **d** Reaction times were more stable in VGPs (N=23) than NVGPs (N=23) in the temporal cue condition. Error bars represent one confidence interval of the mean.

Supplementary Note 3: implicit temporal orienting in sequential effects.

The phenomenon of implicit temporal orienting relies on the automatic expectation of experiencing the same duration as previously experienced (e.g. a trial t_{-1} [7,8]). As a consequence, when the FP at trial *t* is shorter than the FP at trial t_{-1}, implicit temporal expectations are violated and reaction times are delayed (Supplementary Fig. 2). To evaluate this implicit temporal orienting phenomenon, a three-way repeated-measures ANOVA was performed on trials with short FP, with the factors Previous Foreperiod, Cue and Group. Results indicated a main effect of the Previous Foreperiod ($F(1, 44) = 38.79$, $p < .0001$; $\eta^2_p = .469$), such as the reaction times in trials with a short FP were delayed when preceded by a trial with a long FP (Mean = 456ms, CI = 10ms) rather than a short FP (Mean = 345 ms, CI = 10ms). The analysis also revealed a main effect of the Cue ($F(1, 44) = 17.98$, $p = .0001$; $\eta^2_p = .29$), indicating that reaction times for trials with short FP were shorter with the temporal (Mean = 347ms, CI = 11 ms) rather than the neutral cue (Mean = 355 ms, CI = 10 ms). No other effects were reported (all $F(1, 44) < 1.68$, all $p > .202$). Then, we also quantified how much participants were affected by this violation of implicit temporal expectations to control for between-subject variability. For each trial block, we calculated the percentage of speed change given the formula: Speed change (%) = 100 * (Short FP_{t-1} – Long FP_{t-1} / Short FP_{t-1}). A three-way repeated-measures ANOVA was performed on trials with short FP with the factors Block, Cue and Group. No effect was reported (all $F(1, 44) < 2.19$, all $p > .146$). The results replicate the sequential effects of the literature, but the lack of group difference on sequential effects suggests those effects do not originate the group difference on implicit processing.

Supplementary Fig. 2: Sequential effects.

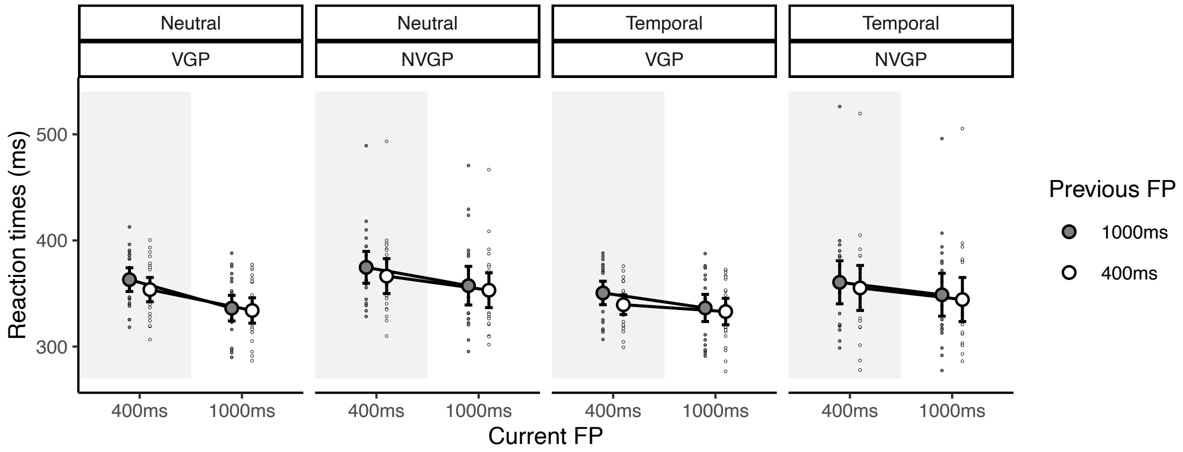

We observe similar sequential effects (shaded areas) between groups and cueing conditions. Error bars represent one confidence interval of the mean.

Supplementary Note 4: Saccade rate inhibition to target anticipation.

We evaluated the inhibition of microsaccades prior to the apparition of the target, reflecting its anticipation [10–14] through the deployment of temporal attention. A two-way repeated-measures ANOVA was performed on the average saccade rates within the 800-1000 ms time-interval in trials with the long FP, with the factors Group and Cue. The analysis revealed an effect of the Cue ($F(1, 44) = 6.53$, $p = .014$; $\eta^2_p = .129$, Supplementary Fig. 3), with a stronger saccadic inhibition in trials with the temporal (Mean = 0.38 Hz, CI = 0.03 Hz) rather the neutral (Mean = 0.44 Hz, CI = 0.04 Hz) cue, supporting the hypothesis that pre-target saccade rates reflect voluntary temporal orienting [12,14,15]. No other effect was reported (all $F(1,44) < 0.37$, all $p > .54$).

Supplementary Fig. 3: Pre-target Saccade Rates (PSR) across cues and groups in trials with long FP.

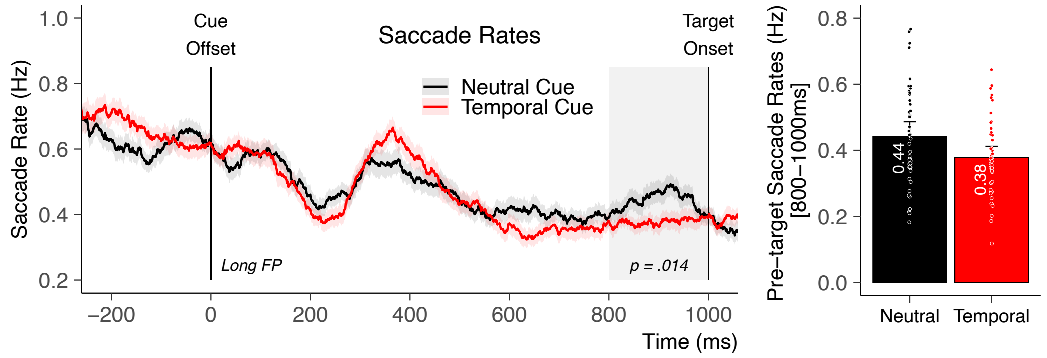

The analysis revealed a main effect of the cue: the saccade rates were more inhibited prior the target onset in trials with the temporal cue (N=46). Coloured shaded areas represent ± one SEM. Error bars represent ± one confidence interval of the mean.

Supplementary Note 5: CNV and oscillatory data analysis.

Visual inspection of the oscillatory activity (Supplementary Fig. 5) supported the selection of the electrode of interest for the analysis of the CNV, theta-band activity and alpha-beta coupling described in the main manuscript. In contrast with the phase-amplitude coupling analysis, during the theta-band analysis no correlation was established between the magnitude of the theta-band oscillations and the benefit from the passage of time in trials with the neutral cue ($p = .36$).

Supplementary Fig. 4: Effect of temporal orienting on the CNV.

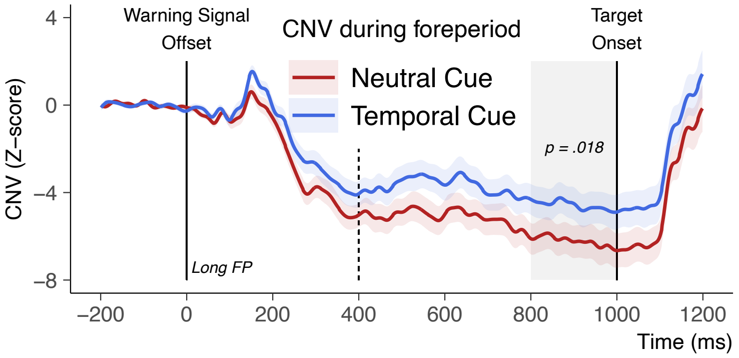

We observe a reduced amplitude of the CNV when the timing of the target is cued. Coloured shaded areas represent ± one SEM.

Supplementary Fig. 5: topographic maps.

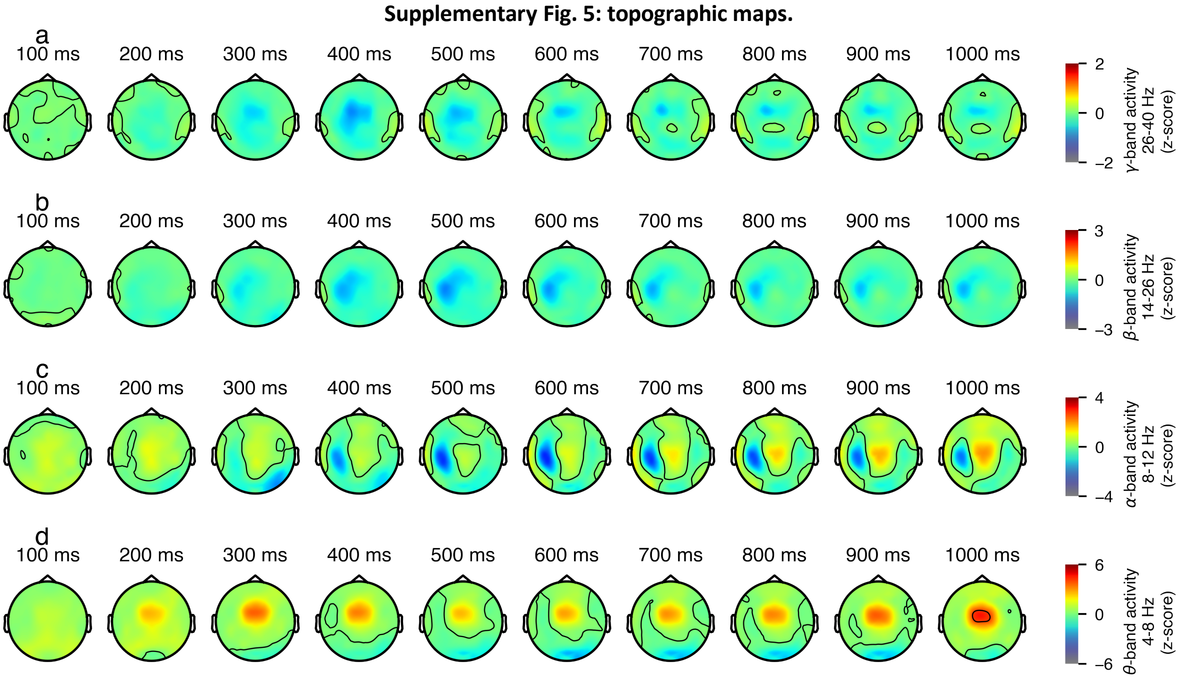

Topographic maps of the averaged gamma (**a**), beta (**b**), alpha (**c**) and theta (**d**) band oscillatory activities in trials with long FP across the cues for all 45 participants. An important reduction of power in the alpha and beta-band was recorded over the left motor cortex. An increase of power in the alpha and theta-band was recorded around the fronto-central electrodes Cz and FCz, respectively.

Supplementary Note 6: **Phase-amplitude coupling (PAC).**

A previous study showed that α-β phase-amplitude coupling (PAC) indexes the temporal precision of self-paced actions [16]. To control for the specificity of the θ-β coupling reported in the main manuscript, we performed non-parametric two-sided Wilcoxon signed-rank tests to evaluate statistical significance. The analysis revealed no evidence of α-β PAC (all $p > .61$). Given the co-existing beta oscillations in both left motor (C3) and frontal median (FCz) cortices, another control analysis evaluated the presence of α-β and θ-β PAC within the FCz. Wilcoxon signed-rank tests indicated no coupling between the theta phase on the amplitude of beta oscillations at FCz (all $p > .68$). Similarly, the analysis revealed no coupling between the alpha phase on the amplitude of beta oscillations at FCz (all $p > .20$).

In addition to the correlation between the θ-β PAC and the benefits from the passage of time, a Spearman correlation was performed to evaluate the relationship between the θ-β PAC and the benefit from the temporal cue. To do so, we correlated the benefit from the temporal cue with the difference of PAC values between the two cueing conditions (Temporal minus Neutral). No relationship was revealed ($r = -.09$, $p = .549$).

Supplementary References

1. Gentile, D. A. *et al.* Internet Gaming Disorder in Children and Adolescents. *Pediatrics* **140**, S81–S85 (2017).
2. Dye, M. W. G., Green, C. S. & Bavelier, D. The development of attention skills in action video game players.

Neuropsychologia **47**, 1780–1789 (2009).
3. Bejjanki, V. R. *et al.* Action video game play facilitates the development of better perceptual templates. *Proc. Natl. Acad. Sci. U. S. A.* **111**, 16961–16966 (2014).
4. Zhang, R. Y. *et al.* Action video game play facilitates "learning to learn". *Commun. Biol.* **4**, (2021).
5. Green, C. S., Pouget, A. & Bavelier, D. Improved probabilistic inference as a general learning mechanism with action video games. *Curr. Biol.* **20**, 1573–1579 (2010).
6. Balasubramaniam, R. *et al.* Neural Encoding and Representation of Time for Sensorimotor Control and Learning. *J. Neurosci.* **41**, 866–872 (2021).
7. Los, S. A., Kruijne, W. & Meeter, M. Hazard versus history: Temporal preparation is driven by past experience. *J. Exp. Psychol. Hum. Percept. Perform.* **43**, 78–88 (2017).
8. Marques-Carneiro, J. E. *et al.* Where and when to look: Sequential effects at the millisecond level. *Attention, Perception, Psychophys.* (2020). doi:10.3758/s13414-020-01995-3
9. Wagenmakers, E.-J. *et al.* Bayesian inference for psychology. Part II: Example applications with JASP. *Psychon. Bull. Rev.* **25**, 58–76 (2018).
10. Denison, R. N., Yuval-Greenberg, S. & Carrasco, M. Directing Voluntary Temporal Attention Increases Fixational Stability. *J. Neurosci.* **39**, 353–363 (2019).
11. Olmos-Solis, K., van Loon, A. M., Los, S. A. & Olivers, C. N. L. Oculomotor measures reveal the temporal dynamics of preparing for search. in 1–23 (2017). doi:10.1016/bs.pbr.2017.07.003
12. Tal-Perry, N. & Yuval-Greenberg, S. Pre-target oculomotor inhibition reflects temporal orienting rather than certainty. *Sci. Rep.* **10**, 1–9 (2020).
13. Amit, R., Abeles, D., Carrasco, M. & Yuval-Greenberg, S. Oculomotor inhibition reflects temporal expectations. *Neuroimage* **184**, 279–292 (2019).
14. Abeles, D., Amit, R., Tal-Perry, N., Carrasco, M. & Yuval-Greenberg, S. Oculomotor inhibition precedes temporally expected auditory targets. *Nat. Commun.* **11**, 1–12 (2020).
15. Badde, S., Myers, C. F., Yuval-Greenberg, S. & Carrasco, M. Oculomotor freezing reflects tactile temporal expectation and aids tactile perception. *Nat. Commun.* **11**, 1–9 (2020).
16. Grabot, L. *et al.* The strength of alpha-beta oscillatory coupling predicts motor timing precision. *J. Neurosci.* **39**, 3277–3291 (2019).
